# Malathion: MAK Value Documentation, addendum – Translation of the German version from 2024

**DOI:** 10.34865/mb12175e11_2ad

**Published:** 2026-06-30

**Authors:** Andrea Hartwig, MAK Commission

**Affiliations:** 1 Institute of Applied Biosciences, Department of Food Chemistry and Toxicology, Karlsruhe Institute of Technology (KIT) Adenauerring 20a, Building 50.41 76131 Karlsruhe Germany; 2 Permanent Senate Commission for the Investigation of Health Hazards of Chemical Compounds in the Work Area, Deutsche Forschungsgemeinschaft, Kennedyallee 40, 53175 Bonn, Germany. Further information: Permanent Senate Commission for the Investigation of Health Hazards of Chemical Compounds in the Work Area | DFG

**Keywords:** malathion, insecticide, pesticide, toxicity, evaluation, acetylcholinesterase inhibitor

## Abstract

Malathion [121-75-5] is not approved in Germany either as a head lice treatment or in pesticides. In the EU the approval is temporarily limited until 2023. The previous MAK Value documentation and addenda do not reflect the current data situation of the substance. The MAK Commissiondecided that a new evaluation is not of high priority. The MAK value and the other classifications are therefore suspended and the substance is listed in the Section II c of the List of MAK and BAT Values for substances no longer evaluated.

**Table:** 

**MAK value**	**see Section II c of the List of MAK and BAT Values**
**Peak limitation**	**–**
	
**Absorption through the skin**	**–**
**Sensitization**	**–**
**Carcinogenicity**	**–**
**Prenatal toxicity**	**–**
**Germ cell mutagenicity**	**–**
	
**BLW (2023)**	**reduction of the acetylcholinesterase activity in erythrocytes to 70% of the reference value^a)^**
	
Synonyms	–
Chemical name (IUPAC)	diethyl 2-dimethoxyphosphinothioylsulfanylbutanedioate
CAS number	121-75-5
Molar mass	330.35 g/mol
Melting point	2.85 °C (IFA 2023)
Boiling point	156–157 °C (IFA 2023)
Density at 25 °C	1.23 g/cm^3^ (IFA 2023)
Vapour pressure	< 0.001 hPa (IFA 2023)
log K_OW_	2.36 (IFA 2023)
Solubility at 20 °C	143 mg/l water (IFA 2023)

^a)^ The BLW (biological guidance value) is derived as the ceiling value because of acute toxic effects.

This addendum was prepared because the previous evaluations no longer reflect the data currently available for the MAK value and for the designations and classifications of the substance. Malathion is a neurotoxic organophosphate that is used to treat head and body lice and for pest control (Greim 2007, available in German only; Singh et al. 2014). It acts as an indirect cholinesterase inhibitor only after activation in the animal organism (Henschler 1973, available in German only). The biological guidance value (BLW) for acetylcholinesterase inhibitors (reduction of the acetylcholinesterase activity in erythrocytes to 70% of the reference value; Lewalter 1995; Weistenhöfer et al. 2024) therefore applies to malathion. The BLW is derived as the ceiling value because of acute toxic effects. However, it was not investigated whether this is the most sensitive end point.

For malathion, a MAK value of 15 mg/m^3^ for the inhalable fraction was derived in 1958 (Henschler 1973). In 2002, the substance was assigned to Peak Limitation Category II with an excursion factor of 4 (Greim 2002). Malathion was classified in Pregnancy Risk Group D in 1992. This classification was confirmed in 2007 (Greim 2007).

Within the EU, the use of malathion in plant protection products is currently permitted until July 31, 2026 in accord­ance with Regulation (EC) 1107/2009 concerning the placing of plant protection products on the market (European Commission 2022 b, 2023) and its use is permitted only in greenhouses with a permanent structure (European Commission 2018). Malathion is listed in Annex I Part 1 of the PIC Regulation (EC) No. 649/2012 and therefore requires an export notification (European Commission 2022 a). Malathion was approved for use as a plant protection product in the Federal Republic of Germany until 1992 and in the former German Democratic Republic until 1994 (BVL 2010). Malathion is not approved in Germany to treat lice in humans or as a veterinary medicinal product (BfArM 2022; UBA 2023).

The previous evaluations (MAK value documentation and addenda) do not reflect the currently available data. However, a re-evaluation of the substance is not a priority. Therefore, the MAK value, the peak limitation and the “H” designation have been withdrawn and malathion has been allocated to Section II c of the List of MAK and BAT Values (DFG 2022). This section lists substances for which the previous MAK values, designations and classifications have been withdrawn and which are no longer being reviewed at present.
